# Efficient Binary Solution Adsorption Using Polyurethane Foam Composites Integrated with Zr-MOF and Milled Activated Carbon

**DOI:** 10.3390/polym18131669

**Published:** 2026-07-06

**Authors:** Supanicha Alapol, Thidarat Imyen, Khemmathin Lueangwattanapong, Nutchapon Chiarasumran, Maythee Saisriyoot, Anusith Thanapimmetha, Yi-Shen Huang, Chih-Feng Huang, Penjit Srinophakun

**Affiliations:** 1 Department of Chemical Engineering, Kasetsart University, Bangkok 10900, Thailand; supanicha.a@ku.th (S.A.); fengtri@ku.ac.th (T.I.); fengkhl@ku.ac.th (K.L.); fengnpc@ku.ac.th (N.C.); fengmts@ku.ac.th (M.S.); fengjrc@gmail.com (A.T.); 2 Department of Chemical Engineering, i-Center for Advanced Science and Technology (iCAST), National Chung Hsing University, Taichung 40227, Taiwan; yishen617@gmail.com; 3 Graduate Program in Semiconductor and Green Technology, Academy of Circular Economy, National Chung Hsing University, Nantou 540216, Taiwan

**Keywords:** polyurethane foam, milled activated carbon, zirconium metal organic framework, hexavalent chromium, Congo red

## Abstract

Wastewater containing heavy metals and dyes poses serious environmental risks. This study developed a multifunctional composite by coating polyurethane foam (PUF) with milled activated carbon (mAC) and a zirconium-based metal–organic framework (Zr-MOF) for the simultaneous removal of hexavalent chromium (Cr(VI)) and Congo red (CR). The composite was synthesized using a hydrothermal method to grow Zr-MOF on the surface. The SEM analysis confirmed the successful incorporation of mAC and surface modification with Zr-MOF, which resulted in increased surface roughness and porous morphology. XRD and FTIR confirmed the presence of organic ligands connected to the metal structure and the functional groups of each component in composite materials. The optimum conditions for Zr-MOF/mAC/PUF adsorption (nearly 100% removal) in the binary Cr(VI)/CR solution (50 mg/L each) were 25 °C, pH 9, and 150 rpm for 24 h. The Zr-MOF/mAC/PUF was hydrophilic with a swelling ratio of 2.64 g/g. The thermodynamic investigation of Zr-MOF/mAC/PUF resulted in 141.6218 kJ/mol for Cr(VI) and 166.111 kJ/mol for CR of ΔH° (rapid adsorption), negative ΔG° (spontaneous adsorption), a high positive value of ΔS° (disorder structure) and low activation energy (approximately 2.5 to 2.8 kJ/mol). After analyzing the isotherm and reaction kinetics, the possible mechanism could be endothermic physicochemical adsorption and pseudo-second-order kinetic behavior, with electrostatic attraction and diffusion control. The study of 6-times-reused Zr-MOF/mAC/PUF adsorption identified as a decrease of 7.55 percentage point without changing notable morphology and functional groups, based on SEM and FTIR.

## 1. Introduction

Heavy metals are used widely in various industries [1]. Wastewater pollutants from industrial activities contain heavy metals, which cause water resource contamination. Therefore, heavy metals can be easily accessible to living organisms and humans through consumption, contact, or contamination, particularly for residents near industrial areas [2,3]. Long-term human health damage has been reported due to the large increases in the use of some heavy metals, such as arsenic (As) and lead (Pb) [4,5]. For example, chromium can trigger allergic reactions in humans, whereas lead can damage the circulatory system and affect the nervous system [6,7,8,9].

Wastewater from dyes is an issue because dyes are widely used in various industries, such as the food, textiles, and paper industries [10] and contain toxic chemicals such as benzidine and other aromatic compounds. The textile industry faces environmental concerns due to the dyeing process and finishing operations, as 17–20% of total industrial water pollution comes from textile dyeing factories [11]. Globally, synthetic dyes are popular and used widely, with over 10,000 types or approximately 700,000 tonnes of synthetic dyes used annually [11]. Chemicals used in the textile production process, such as dyes and fixative agents that contain chromium, lead, cadmium, and other heavy metals, are removed from factory processing lines into the wastewater system. Approximately 280,000 tonnes of these dyes are released into surface water due to inefficient wastewater treatment, which has received severe criticism [12,13]. Dyes in wastewater can block the sunlight, reduce the oxygen level, and interrupt ecosystems [11,12]. Congo red (CR) dye has been used intensively in the production of jeans and textiles. It is classified as a carcinogen because of the presence of stable aromatic rings in the CR molecular structure, made up of aromatic amine groups, making it highly resistant to biodegradation and allowing it to persist in the environment for weeks or months. Therefore, eliminating Congo red in wastewater is essential for environmental protection [13].

Polyurethane foam (PUF), made from isocyanate and polyols, such as diamine or diols, is a synthetic polymer which is used in many applications. In addition, its properties and cell structure can be improved by adding various substances such as blowing agents, surfactants, and catalysts [13,14]. Due to the presence of both polar and non-polar functional groups on the surface of PUF, it can retain aromatic compounds, metallic dithionates, or large anions [14,15,16]. PUF is attractive due to its high porosity, low cost, and easy functionalization, enabling the removal of metals, dyes, and organic pollutants [17]. Currently, the development of PUF is focused on progressing from basic materials to advanced functional materials. Its structure and properties are improved through the addition of nanomaterials, active ingredient coatings, and integration with high-surface-area materials such as MOFs and modified carbon. This enhances adsorption capability, contaminant specificity, antimicrobial properties, and service durability. As a result, PUF can be applied to highly efficient water treatment systems, handle complex pollutant removal, and offer improved reuse capabilities on an industrial scale [18,19].

Activated carbon (AC) is used in a wide range of industrial applications, including food processing, pharmaceuticals, chemicals, petroleum, mining, nuclear, automotive, and vacuum production [14]. AC is produced by chemical and physical activation processes that create pores and modify the surface of raw materials. It has low density due to its high porosity and surface area. Therefore, AC is used commonly for gas and liquid adsorption. The size of the AC affects adsorption efficiency. Many researchers have found that milled activated carbon (mAC) has excellent adsorption capabilities for all types of inorganic substances [14,15,16]. It is a widely available, environment-friendly, and recyclable material. AC derived from biomass has been tested and reported to remove various heavy metals from aqueous solutions, improving the quality of the treated water. It is easy to use and resistant to multiple toxins [16,20].

In the industrial sector, zirconium-based MOF (Zr-MOF) is used as a technology for the adsorption of Cr(VI) and CR. Zr-MOF, namely UiO-66-(COOH)_2_, is of interest as a highly efficient adsorbent due to its specific surface area, abundant reactive metal sites, and –COOH groups. In particular, the pendant –COOH groups provide additional active sites for interaction with pollutant species and have been reported to enhance the adsorption affinity of UiO-66-type materials toward metal ions and charged contaminants through electrostatic interactions, hydrogen bonding, and surface complexation [21,22,23]. Furthermore, UiO-66-(COOH)_2_ exhibits excellent chemical stability in aqueous environments, which is advantageous for wastewater treatment applications. The combination of high stability, porosity, and the abundant free carboxyl functional groups motivated the selection of UiO-66-(COOH)_2_ for the removal of Cr(VI) and Congo red in this study.

This research focuses on the synthesis of PUF with AC and a modified surface involving a Zr-MOF precursor to create a new material, Zr-MOF/mAC/PUF, due to the limitations of existing materials, such as PUF, which has relatively low adsorption capacity and specificity, and AC, which, despite its high surface area, lacks specificity and efficiency in removing mixed contaminants; as well as limitations in the reuse and stability of composite materials in other studies, the need for developing new materials has been highlighted. The Zr-MOF/mAC/PUF composite material is highly suitable because it combines the advantages of each component. PUF acts as a highly porous support structure that facilitates the flow of the solution. The improved adsorption performance may be associated with the incorporation of mAC and Zr-MOF. As a result, this material has the potential to enhance adsorption efficiency, target specificity, and reuse, making it suitable for development as a novel material for complex industrial water treatment.

This study compared the adsorption efficiency of PUF, mAC/PUF, Zr-MOF/PUF, and Zr-MOF/mAC/PUF in binary pollutant solutions and their physical and thermal stability. In addition, the Zr-MOF/mAC/PUF composite material developed in this research has the potential to advance research in adsorbent materials for wastewater treatment. Previous research has focused mainly on investigating binary adsorption systems using Zr-MOF-based materials [21]. Limited attention has been given to the development of structured monolithic composites integrating polyurethane foam, AC, and Zr-MOF for simultaneous adsorption applications. Therefore, this study focuses on the fabrication of a reusable Zr-MOF/mAC/PUF composite with enhanced structural stability and adsorption functionality for wastewater adsorption. This research presents an adsorption study in a binary system of Cr(VI) and CR, which better reflects real wastewater conditions of textile and leather tanning, which were more complex and have largely been ignored in other published research. By developing Zr-MOF/mAC/PUF composite materials, we can combine the advantages of three materials: the porous structure and flexibility of PUF, the high surface area of mAC, and the specific active site of Zr- MOF. The adsorption parameters were the initial concentration, temperature, and pH of the solutions. Subsequently, adsorption isotherms, kinetic isotherms, and thermodynamic models were investigated to understand the interaction behavior between the adsorbent and adsorbate. Finally, the reusability of the materials was examined.

## 2. Materials and Methods

### 2.1. Chemicals

Coconut shell-derived AC was purchased from Henan Xingnuo Environmental Protection Materials Co., Ltd. (Zhengzhou, China). Polyethylene glycol (PEG2000, MW = 2000 g/mol) was obtained from Tokyo Chemical Industry Co., Ltd. (Fukaya, Japan). Polydimethylsiloxane (PDMS, MW 4200 g/mol) was a product of the Shepherd Chemical Company (Cincinnati, OH, USA). Tin (II) 2-ethylhexanoate (C_16_H_30_O_4_Sn; stannous octoate) was purchased from Thermo Fisher Scientific (Waltham, MA, USA). Glycerol (A.R., 99.5%) was obtained from the QReC (Brisbane, Australia). Methylenebis (phenyl isocyanate; 98%) was purchased from Alfa Chemical Co., Ltd. (Heppenheim, Germany). Potassium dichromate (K_2_Cr_2_O_7_), Congo red (C_32_H_22_N_6_Na_2_O_6_S_2_), sodium hydroxide (NaOH, pellets, 97.0%), and sodium chloride (NaCl, 99%) were supplied by KemAus™ (Cherrybrook, Australia). Zirconium chloride (ZrCl_4_) was obtained from Sigma-Aldrich France (Saint-Quentin-Fallavier, France), and 1,2,4,5-benzenetetracarboxylic acid (H_4_BTEC) was purchased from Sigma-Aldrich (Schnelldorf, Germany). All reagents were used as received, without further purification.

### 2.2. Activated Carbon Preparation

The AC (10 g) was milled using zirconium balls in a jar ball mill (Tai Yiaeh; Taipei, Taiwan) at a rotation speed of 150 rpm for 1 h. Then, the milled activated carbon (mAC) was produced following sieving (Retsch Test Sieve Shaker) at 80 mesh. The amount of mAC, 0.32 g or 1.1 wt%., was mixed with PUF for the mAC/PUF synthesis.

### 2.3. Preparation of Zr-MOF Precursor

A zirconium-based metal–organic framework (Zr-MOF) was synthesized via a hydrothermal reaction between ZrCl_4_ and pyromellitic acid (H_4_BTEC). Throughout this work, the material is referred to as a Zr-MOF or UiO-66-(COOH)_2_ to reflect its composition. The Zr-MOF nanoparticles were prepared according to the procedure reported by [23]. At first, 1.16 g ZrCl_4_ and 1.08 g H_4_BTEC were dispersed in 50 mL of deionized water using a stirrer (300 rpm) at room temperature for 3 h. Then, the solution was heated at 120 °C for 24 h. The as-obtained white gel was centrifuged at 1000 rpm for 10 min and washed with deionized water until neutral.

### 2.4. PUF Synthesis

The pure PUF was synthesized by mixing 18 g of PEG2000, 9 g of MDI, 0.6 mL of PDMS, 0.6 mL of glycerol, and 1.5 mL of tin (II) 2-ethylhexanoate (C_16_H_30_O_4_Sn; stannous octoate). First, PEG2000, PDMS, and glycerol were mixed at 1000 rpm in a thermoresistant plastic beaker. Then, the MDI was added and stirred at 1000 rpm for 5 s. Next, the stannous octoate was added and stirred for 12 s. Then, the mixture was allowed to stand for 24 h at room temperature to ensure complete curing [24]. The foam was removed from the beaker the next day and cut to a size of 1 × 1 × 1 cm. For the mAC/PUF synthesis, the process followed the same steps as above for the PUF synthesis, except that 0.32 g of mAC was added in the mixing step of PDMS and glycerol before foaming.

### 2.5. Synthesis of Zr-MOF/PUF and Zr-MOF/mAC/PUF

The hydrothermal method was used for the fabrication of Zr-MOF/PUF and Zr-MOF/mAC/PUF. In brief, 4 g each of PUF and mAC/PUF were placed in a Teflon-lined reaction vessel. Then, 80 mL of Zr-MOF precursor solution was added. The reaction was carried out in an oven (model UF 55; Memmert; Pathum Thani, Thailand) at 120 °C for 24 h. After the reaction, the composite materials were cooled at room temperature. The obtained Zr-MOF/PUF and Zr-MOF/mAC/PUF were washed with deionized water to remove any loose powder from their surfaces. Finally, all composite foams were dried at 60 °C for 12 h.

### 2.6. Characterization of Synthesized Composites

The synthesized composites were characterized using X-ray diffractometry (XRD), scanning electron microscopy (SEM), and Fourier-transform infrared (FTIR) spectroscopy. The thermal stability of the composite materials was analyzed using high-temperature thermogravimetric analysis (TGA) in a heating range up to 700 °C with a temperature gradient of 10 °C/10 min in nitrogen. Finally, the water contact angle was determined (Dino-Lite Digital Microscope; Bangkok, Thailand) and the swelling ratio of all composites was determined. Please note that the Brunauer–Emmett–Teller (BET) method could not be applied to these composite materials because the soft sponge PUF had collapsed under vacuum, but the pore sizes were estimated from SEM.

### 2.7. Preparation of Binary Solutions

Binary solutions were prepared by dissolving 10–50 mg of Cr(VI) and CR (according to the desired concentrations) in 1 L of distilled water and mixing thoroughly for 30 min. Sodium hydroxide (0.1 mol/L) and concentrated hydrochloric acid (37%) were used to adjust the pH to 4, 7, and 9.

### 2.8. Batch Adsorption Experimental Set-Up

Batch adsorptions were carried out using 4 g of adsorbent and 100 mL of binary Cr(VI)/CR solution in a 250 mL Erlenmeyer flask. All experiments took place in an incubator (Model SI2; CT Laboratory Co., Ltd., Bangkok, Thailand) at a constant agitation rate of 150 rpm for 24 h with different pH (4, 7, 9), temperature (25, 35, 45 °C), and initial concentration of Cr(VI) and CR (10, 20, 30, 40, 50 mg/L). After adsorption, samples were taken every 2 h in triplicate. The concentration of Cr(VI) in the solution was measured using ultraviolet-visible spectrophotometry at 365 nm [25,26]. The concentration of CR dye was determined based on visible spectroscopy at wavelengths of 495 nm under alkaline conditions and at 565 nm under acidic conditions [27]. The removal percentages of Cr(VI) and CR were calculated using Equations (1)–(3).

(1) $$\%Removal\;of\;Cr(VI)=\frac{C_{0\left(365\;\mathrm{nm}\right)}-{\;C}_{t(365\;\mathrm{nm})}}{C_{0\left(365\;\mathrm{nm}\right)}}\;\times \;100$$

(2) $$\%Removal\;of\;CR\;(pH\geq 7)=\frac{C_{0\left(495\;\mathrm{nm}\right)}-C_{t(495\;\mathrm{nm})}}{C_{0\left(495\;\mathrm{nm}\right)}}\;\times \;100$$

(3) $$\%Removal\;of\;CR\;(pH<7)=\frac{C_{0\left(565\;\mathrm{nm}\right)\;}-{\;C}_{t(565\;\mathrm{nm})}}{C_{0\left(565\;\mathrm{nm}\right)}}\;\times \;100$$

where C_0_ and C_t_ are the initial and end adsorption concentrations of Cr(VI)/CR in the solution at equilibrium, respectively.

The thermodynamics for the Cr(VI) and CR adsorption were described by Equations (4)–(7).

(4) $$\;K_{d}=\frac{C_{i}-C_{e}}{C_{e}}$$

(5) $${\mathrm{lnK}}_{d}=-\left(\frac{∆H{}^{\circ}}{\mathrm{RT}}\right)+(\frac{∆S{}^{\circ}}{R})$$

(6) ΔG°=∆H°−TΔS°

ΔH° = E_a_ − RT (7)

where K_d_ is the distribution coefficient for Cr(VI)/CR adsorption, C_i_ is the initial concentration, C_e_ is the equilibrium concentration, ∆H° is the enthalpy change, ∆G° is the Gibbs energy change, ∆S° is the entropy change, E_a_ is the activation energy, R is the universal gas constant, and T is the absolute temperature in Kelvin.

The data obtained were examined according to two adsorption isotherms, namely, Langmuir and the Freundlich [28]. The Langmuir model was selected to estimate the maximum adsorption capacity correlated with monolayer coverage on the adsorbent surface and shown in Equation (8).

(8) $$C_{e}/q_{e}=1/K_{L}q_{max}+\frac{C_{e}}{q_{\max}}$$

where q_max_ (mg/g) is the maximum monolayer adsorption capacity, K_L_ (L/mg) is the Langmuir constant related to adsorption energy, q_e_ (mg/g) is the equilibrium adsorption capacity, and C_e_ (mg/L) is the equilibrium concentration. The Freundlich model describes multilayer adsorption on a heterogeneous surface, as shown in Equations (9) and (10).

logq_e_ = logK_f_ + 1/n (logC_e_) (9)

(10) $$q_{e}=K_{f}C_{e}^{1/n}$$

where K_f_ (L/mg) is the adsorption capacity and n is the adsorption intensity or surface heterogeneity.

The pseudo-first-order model is associated with adsorption processes controlled by molecular diffusion, known as physisorption, and with relatively weak surface interactions. Often, the pseudo-second-order model is associated with chemisorption or charge-exchange interactions between the adsorbent and the solution, as shown by Equation (11).

ln (q_e_ − q_t_) = lnq_e_ − K_1_t (11)

where q_e_ (mg/g) is the adsorption capacity at equilibrium, q_t_ (mg/g) is the adsorption capacity at time (t), and K_1_ (min^−1^) is the pseudo-first-order rate constant.

Equation (12) presents the form for a pseudo-second-order rate constant.

t/q_t_ = 1/K_2_q_e_^2^ + t/q_e_ (12)

where K_2_ (min^−1^) is the pseudo-second-order rate constant.

The swelling ratio (g/g) of the four materials versus time (min) was calculated using Equation (13):

(13) $$Swelling\;ratio=\frac{W_{\mathrm{wet}}\;-{\;W}_{\mathrm{dry}}}{W_{\mathrm{dry}}}$$

where W_dry_ is the mass of the material in a dry state and W_wet_ is the mass after water adsorption until equilibrium is reached. The difference between these two values represents the amount of water absorbed; dividing by the dry weight gives the swelling ratio of the material.

## 3. Results

The synthesis of the polyurethane foam (PUF) incorporated with milled activated carbon (mAC) and coated with Zr-MOF via a hydrothermal method was conducted to enhance the adsorption performance for Cr(VI) and Congo red (CR) in a binary solution system. Characterization of physical and thermal properties is essential to elucidate the adsorption mechanisms and to confirm the successful synthesis of the composite material. Adsorption experiments require the control of key parameters, including initial concentration, temperature, and pH, in order to systematically evaluate adsorption behavior. Then, the obtained data can be interpreted using the adsorption models to compare the performance of the four different adsorbents: PUF, mAC/PUF, Zr-MOF/PUF, and Zr MOF/mAC/PUF.

### 3.1. Characterization of PUF and PUF Composites

#### 3.1.1. Surface Characteristics of the Synthesized Adsorbents

**Figure 1:**
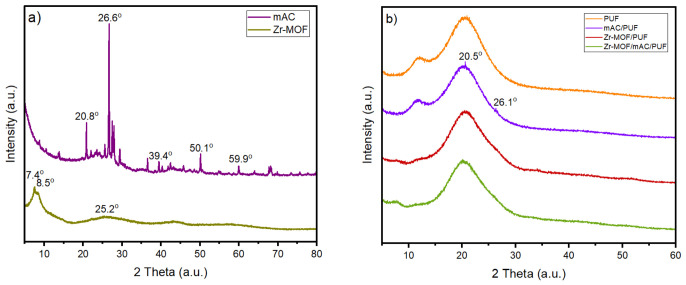
(**a**) XRD patterns of Zr-MOF and mAC and (**b**) XRD patterns of pure PUF and composites.

As shown in Figure 1a, the XRD pattern of pure Zr-MOF had diffraction peaks at 7.4° and 8.5°, consistent with those reported elsewhere [23]. However, the weak intensity and the broad peak centered at 25.2° indicated that the synthesized Zr-MOF had low crystallinity. For the composites, the XRD patterns were dominated by a broad diffraction feature centered at approximately 20–21° (Figure 1b), characteristic of the largely amorphous PUF matrix. Although Zr-MOF and mAC were incorporated into the PUF, their characteristic diffraction peaks were not clearly observed in the composite patterns. This may be attributed to overlaps between the broad amorphous halo of PUF and the characteristic reflections of the Zr-MOF and mAC, or to the high dispersion of the Zr-MOF and mAC particles within the polymer network.

**Figure 2:**
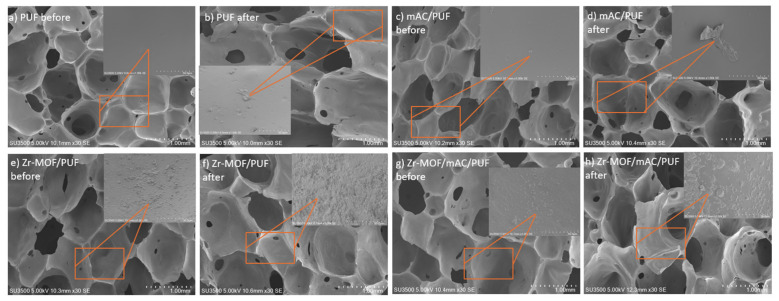
Morphological characteristics of composites before and after adsorption of Cr(VI)/CR solution (50 mg/L, pH 9, 25 °C, 24 h): (**a**,**b**) PUF, (**c**,**d**) mAC/PUF, (**e**,**f**) Zr-MOF/PUF, and (**g**,**h**) Zr-MOF/mAC/PUF.

All experiments were carried out using Cr(VI)/CR solution (initial concentration of 50 mg/L each) at a constant pH of 9 and 25 °C for 24 h. The estimated pore sizes of PUF, mAC/PUF, Zr-MOF/PUF, and Zr-MOF/mAC/PUF are 1.62, 0.8, 0.89, and 0.97 µm, referring to SEM analysis. Adding mAC or Zr-MOF made the PUF pore narrower. Initially, the PUF surfaces were smooth, as shown in Figure 2a, compared with mAC/PUF, where the mAC particles were spread across the PUF surface, as shown in Figure 2c. Promisingly, after adding mAC, the surface of the mAC/PUF showed increased roughness to aid adsorption. However, after adsorption for 24 h, surface damage was observed on both materials. The PUF had roughness around the surface, illustrating structural damage after adsorption (Figure 2b), whereas adding AC helped to reduce swelling and wiping that resulted in the surface sustaining only minor damage (Figure 2d) [29]. AC contains various functional groups capable of binding metals, such as carboxyl (–COOH), lactone, phenolic –OH, carbonyl (C=O), and ether groups, as well as aromatic π-π electron structures [30]. These functional groups provide more active sites for binding metal ions and negatively charged dye molecules on the surface of materials. Therefore, mAC in the materials reduced the noticeable damage and increased the expansion of PUF, allowing the surface to adsorb sufficient volume, preventing damage and abrasion to the surrounding area [31,32]. Zr-MOF/PUF and Zr-MOF/mAC/PUF before adsorption are shown in Figure 2e,g, showing the surface coated with Zr-MOF and mAC particles on the surface of the Zr-MOF/mAC/PUF composites, due to the addition of a functional group of Zr-O, which increased adsorption from the attractive force between the Cr(VI)/CR functional group, a role confirmed by the FTIR. After adsorption, the Zr-MOF/PUF surface peeled off due to a limited area for solution adsorption, as shown in Figure 2f, causing the PUF surface to flake. In the comparison between Zr-MOF/PUF and Zr-MOF/mAC/PUF after adsorption, the surface area of the added mAC materials was retained better due to mAC, which reduced damage to the surrounding adsorption surface, as shown in Figure 2h. Figure 2g shows the promising porous structure of Zr-MOF/mAC/PUF and the successfully loaded mAC and added Zr-MOF on PUF for efficiency and sustainability for the adsorption of Cr(VI)/CR [33].

**Figure 3:**
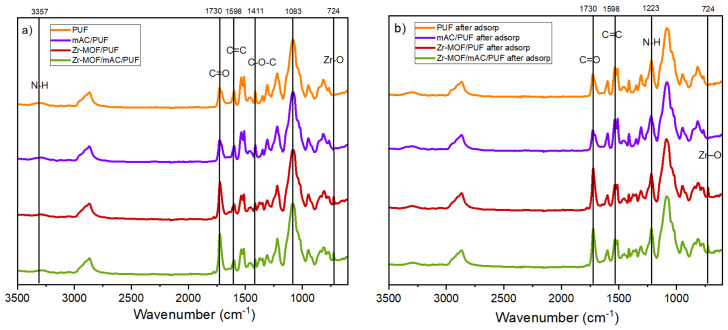
FTIR spectra of PUF, mAC/PUF, Zr-MOF/PUF, and Zr-MOF/mAC/PUF (**a**) before and (**b**) after absorption of Cr(VI)/CR.

**Figure 4:**
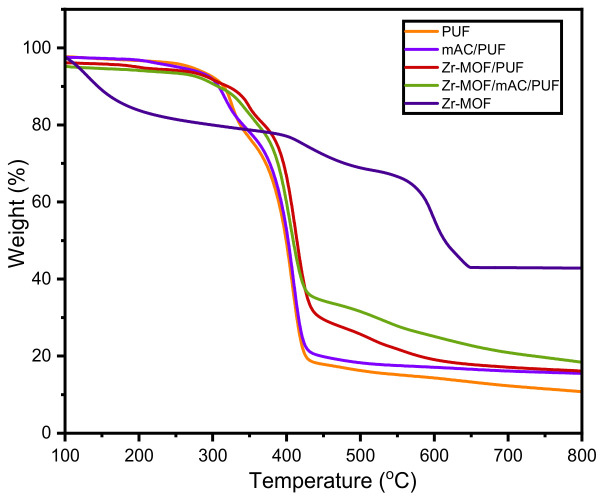
TGA curves of the PUF, mAC/PUF, Zr-MOF/PUF, Zr-MOF/mAC/PUF, and Zr-MOF.

Functional group analysis was conducted to investigate the chemical structures and functional groups on the surfaces of the adsorbent materials. The functional groups of the materials were obtained both before and after adsorption. FTIR spectroscopy was used to measure wavenumbers in the range 600–3500 cm^−1^, with PUF before and after adsorption being observed at 1070–1132 cm^−1^, 3357 cm^−1^, and 1730 cm^−1^, which could be identified as C–O, N–H, and C=O bonds, respectively. At around 1598 cm^−1^, the intensity of the peak corresponding to the C=C bond stability in the PUF as shown in Figure 3a and mAC/PUF samples before and after adsorption indicated that the -NCO groups of MDI (an ingredient in the PUF synthesis) reacted with the hydroxyl (-OH) groups on the mAC/PUF surface via -NCO and -OH reactions, forming carbamate bonds [34] and the Zr–O bond at an intensity of 724 cm^−1^. There may have been physical and covalent bonding between the PUF and AC, which made the mAC/PUF highly thermal resistant (see the TGA profile in Figure 4). Physical bonding occurs from the oxygen-containing functional groups on the surface of AC and the amine and carbonyl groups of PUF material. On the other hand, the isocyanate groups in the polyurethane prepolymer can react directly with the hydroxyl groups on the AC surface to form chemical bonding. In addition, the presence of C=O, -OH, and benzene ring peaks confirmed the existence of functional groups from organic ligands connected to the metal structure [23]. After adsorption of the Cr(VI)/CR solution, the peaks showing no marked changes corresponded to -OH, -NH, and C–O groups, as shown in Figure 3b, confirming that after adsorption of a highly concentrated solution, the material maintained its perfect pore structure (as confirmed by the SEM images) and retained its functional groups that effectively performed adsorption. Cr(VI) adsorption indicated the formation of bonds between the metal ions and functional groups on the material surface, particularly metal–oxygen coordination [23]. Heavy metal adsorption occurred through complex interactions between the metal ions and the functional groups of the Zr-MOF/PUF and Zr-MOF/mAC/PUF composites. As previously mentioned, the incorporation of mAC into PUF led to chemical interactions forming carbamate linkages and further facilitated interactions with Zr–O bonds. The combination of mAC and Zr-MOF in the PUF matrix enhanced the availability of functional groups for adsorption, thereby improving the adsorption efficiency of the adsorbent materials.

#### 3.1.2. Thermal Stability of Composites

TGA was used to assess the thermal degradation temperatures of PUF, mAC/PUF, Zr-MOF/PUF, and Zr-MOF/mAC/PUF samples, as shown in Figure 4. The thermal decomposition behavior of the PUF sample was characterized by two main degradation stages. The first stage began at approximately 25 °C, with the maximum degradation rate at 300 °C corresponding to the decomposition of the -NCO bond in the PUF [35]. The second stage was above 356 °C, which was associated with the degradation of the -OH group [36], representing the primary degradation process of PEG. Comparing the thermal stability curves of PUF and mAC/PUF, pure PUF had lower thermal stability than mAC/PUF. Furthermore, interactions between mAC particles and functional groups on the PUF chains improved thermal stability by restricting chain mobility during the initial decomposition stage and thereby limiting the thermal degradation of urethane bonds. Additionally, mAC trapped free radicals generated during thermal degradation, retarding polymer chain decomposition. The thermal decomposition of Zr-MOF, Zr-MOF/PUF, and Zr-MOF/mAC/PUF occurred in three main stages. The first stage, below 200 °C, was attributed to the evaporation of crystallized water within the pores. The second stage, between 200 and 350 °C, was due to the loss of residual solvent [37]. The third stage, between 351 and 600 °C, corresponded to the decomposition of the organic linker H_4_BTEC within the Zr-MOF framework [14]. The incorporation of Zr-MOF into the composite enhanced overall thermal stability because the -OH groups and Zr^4+^ metal centers could form hydrogen bonds or coordination interactions with the -NCO or -OH groups of PUF [33]. These interactions strengthened the polymer network, restricted chain movement, and increased the onset temperature of thermal degradation [38].

The TGA profiles of Zr-MOF, Zr-MOF/PUF, and Zr-MOF/mAC/PUF had third-step weight losses of 25.18, 11.59, and 14.43%, respectively, as detailed in Table 1. Dehydroxylation of zirconium clusters and the carboxylate ligand was evidenced between 257 and 537 °C. Figure 4 and Table 1 show that adding AC to PUF improved thermal stability. The incorporation of mAC into the PUF structure was approximately 1.1 wt%. Similarly, Zr-MOF/mAC/PUF was more stable than Zr-MOF/PUF. However, Zr-MOF/PUF had better thermal stability than mAC/PUF, probably because the metal–organic framework structure acted as a barrier to protect the polymer chains in PUF [33]. Furthermore, the amount of Zr-MOF in the matrix could be calculated from the third degradation stage of the Zr-MOF/PUF and Zr-MOF/mAC/PUF adsorbents. As the third degradation stage was a Zr-MOF structure, the amount of Zr-MOF loaded into the matrix was in the range 11.59 wt%.

### 3.2. The Surface and Structural Properties of PUF and PUF Composites

#### 3.2.1. Water Contact Angle

**Figure 5:**
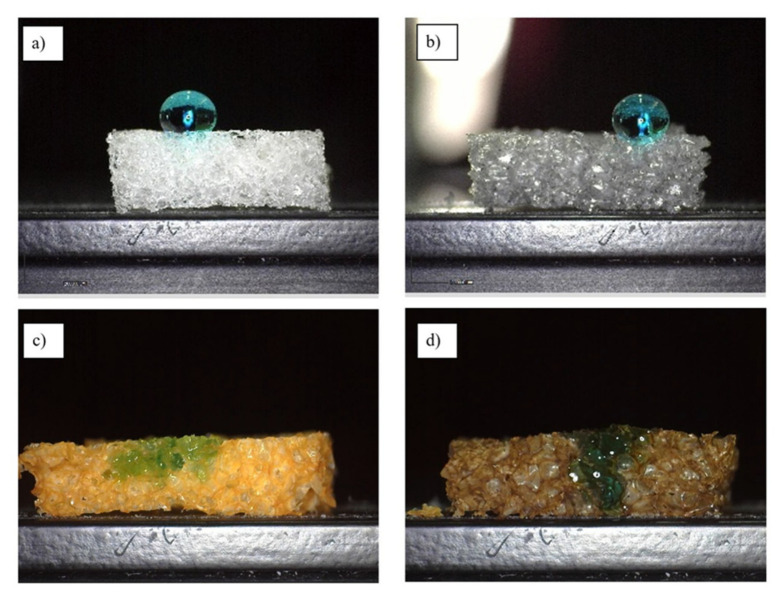
Water contact angle of (**a**) PUF, (**b**) mAC/PUF, (**c**) Zr-MOF/PUF, and (**d**) Zr-MOF/mAC/PUF.

The water contact angle measures the hydrophilicity and hydrophobicity of materials. As shown in Figure 5a, the PUF samples had a contact angle of 101° when mAC was incorporated into the PUF matrix, which reduced their hydrophobicity compared to PUF. Mostly, mAC particles were embedded within the PUF matrix (mAC/PUF material, as shown in Figure 5b). Numerous surface ridges were observed, which were favorable for good adsorption properties [39]. Figure 5c,d indicate that Zr-MOF composites exhibited hydrophilicity. When a water droplet was placed on the surface for measurement, it was immediately absorbed into the foam structure. Zr-MOF/mAC/PUF can absorb more water into the pores of the material than Zr-MOF/PUF. Zr-MOF/mAC/PUF had higher efficiency in absorbing heavy metals and dyes, with the hydrophilicity increasing sustainability. Hydrophobic properties occur preferentially at hydrophilic adsorption sites. This provided evidence of the hydrophilic surface properties of the material, supporting the hypothesis that Zr-MOF enhanced the hydrophilicity of the composite [40].

#### 3.2.2. Swelling Test

**Figure 6:**
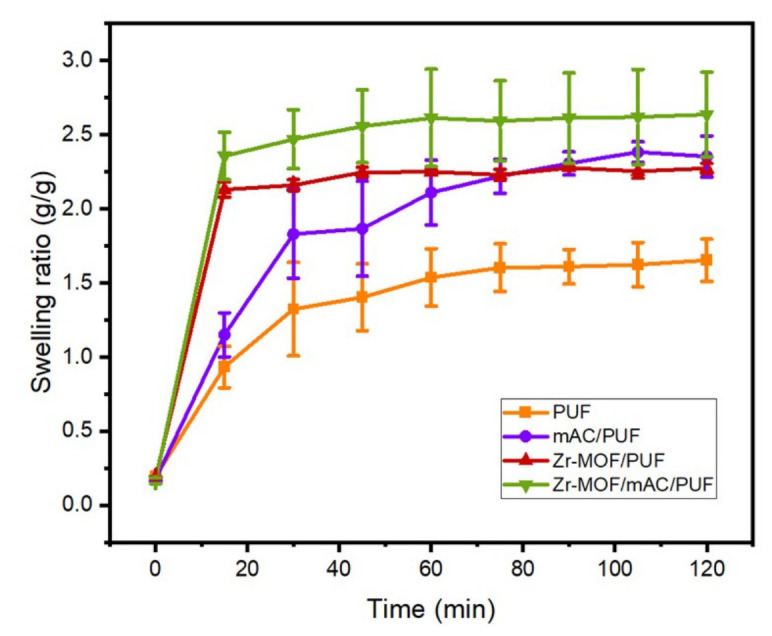
Swelling ratio during adsorption time of PUF, mAC/PUF, Zr-MOF/PUF, and Zr-MOF/mAC/PUF.

Swelling tests of the composite materials were conducted to evaluate their water adsorption capacity and structural stability in solutions. Composite materials were immersed in distilled water at room temperature for 120 min, as shown in Figure 6. Excess water on the surface of the composite materials was removed carefully. Then, the samples were weighed until a constant weight was reached. Figure 6 shows that Zr-MOF improved liquid uptake and swelling behavior. The PUF sample had the lowest swelling among the materials, with a rapid increase at the beginning and a constant value after about 60 min, indicating that the foam itself had a limited adsorption capacity. The mAC/PUF samples had higher swellings than the PUF sample, but remained lower than that of the Zr-MOF composites. The addition of mAC substantially increased the adsorption performance and the adsorption capacity. Zr-MOF/PUF had a considerably higher swelling ratio than PUF and mAC/PUF, with a rapid increase at the beginning and a constant value thereafter. Zr-MOF/mAC/PUF had the highest swelling ratio, which increased continuously and reached a maximum level, indicating that the combination of mAC and Zr-MOF in the PUF matrix created a synergistic effect, maximizing adsorption efficiency.

### 3.3. Parameter Optimization

#### 3.3.1. Spectra of Cr(VI), CR and Binary Systems

**Figure 7:**
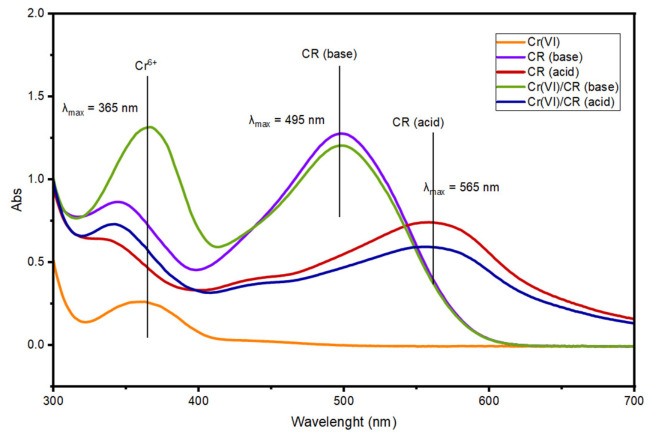
Spectra of Cr(VI), CR in forms of alkaline and acidic pH and Cr(VI)/CR in binary solution (initial concentration = 20 mg/L).

The determination of λ_max_ values for Cr(VI) and CR is an essential step that enables accurate evaluation of the adsorption performance of the adsorbent for Cr(VI)/CR mixtures. Figure 7 shows the absorption spectra of Cr(VI) under alkaline and acidic conditions, with peaks at 365 nm and 565 nm, and the spectrum of CR with a peak at 495 nm. These characteristic peaks are crucial as indicators for monitoring the concentration changes of each component in mixed systems. Even in binary systems, containing both Cr(VI) and CR, the characteristic peaks remained clearly distinguishable without substantial overlap, indicating that UV–Vis spectroscopy was a reliable method for analyzing and tracking the removal efficiency of each component in the binary system.

#### 3.3.2. Effects of Initial Concentration and Adsorption Isotherm

**Figure 8:**
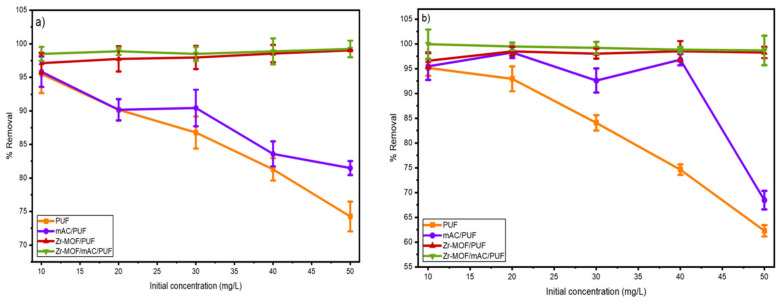
(**a**) Effect of initial concentration of Cr(VI) adsorption, (**b**) effect of initial concentration of CR.

Adsorption isotherm models were used to describe the equilibrium interaction between the Cr(VI)/CR molecules and the adsorbents. Among the several models tested, the Langmuir and the Freundlich isotherms were selected because they best represent mechanisms commonly observed in solid-liquid systems [41]. The percentage of Cr(VI)/CR adsorption on PUF, mAC/PUF, Zr-MOF/PUF, and Zr-MOF/mAC/PUF adsorbents was studied at initial concentrations in the range 10–50 mg/L, as shown in Figure 8a,b. The percentage of removal of Zr-MOF/mAC/PUF reached an efficiency of nearly 99% for all initial concentrations. Table 2 summarizes the calculated parameters. The coefficient of determination of CR on PUF (R^2^ of 0.9994) and mAC/PUF (R^2^ of 0.9928) was high, implying monolayer behavior on homogeneous surfaces of the Langmuir model [42]. However, it was not the case for other absorbents. Low coefficients included K_L_ and R_L_. K_L_ was negative, indicating that the Langmuir model did not adequately fit the experimental data for Zr-MOF/mAC/PUF adsorption.

The Freundlich model provided a better fit, particularly for Zr-MOF/mAC/PUF with CR adsorption, suggesting better adsorption on heterogeneous surfaces with multiple active sites. Zr-MOF and mAC had increased numbers of active sites, reduced pore sizes (estimated by SEM), and improved adsorption performance of the PUF matrix, supporting the adsorption of ionic and organic pollutants [43,44]. The Freundlich constant (K_f_), representing adsorption capacity, increased from 0.34 for PUF to 2.66 for Zr-MOF/mAC/PUF for Cr(VI) adsorption, confirming multilayer adsorption behavior. For CR adsorption, K_f_ ranged from 0.35 for PUF to 1.31 of Zr-MOF/mAC/PUF. These increases confirmed that the modified surface with Zr-MOF and added mAC improved surface heterogeneity and the number of available binding sites. Furthermore, n varied depending on the adsorbent and pollutant type. Several systems had n values lower than 1, indicating heterogeneous adsorption behavior and relatively weak adsorption affinity, suggesting favorable multilayer adsorption driven by the coexistence of electrostatic attraction, hydrogen bonding, and π–π stacking interactions between dye molecules and the composite surface [45]. However, those linearized isotherms might not be sufficient to fit our data and may become a limitation of this study. A non-linear isotherm, such as the Sips model, was suggested for surface heterogeneity in future studies.

#### 3.3.3. Effect of Temperature and Thermodynamic Study

During the adsorption process, temperature plays a vital role in driving the diffusion of Cr(VI) and CR molecules into adsorbent materials. The thermodynamic parameters, namely, Gibbs free energy change (ΔG°), enthalpy change (ΔH°), entropy change (ΔS°), and activation energy (E_a_), were determined to evaluate the feasibility, spontaneity, and energetics. Table 3 presents thermodynamic parameters for Cr(VI) and CR binary solution adsorption on different adsorbents. In general, the values of ΔG°, ΔH°, and ΔS° of all absorbents in Table 3 are in the same trend. Negative values of ΔG° mean spontaneous at operational temperatures. High positive values of ΔS° indicate that ΔS° increases disorder in the structure. This evidence suggested that the Cr(VI) and CR molecules moved into adsorption sites on the surface of the adsorbent materials, rearranging the surrounding water molecules and other nearby molecules. The highest entropy value was observed for Zr-MOF/mAC/PUF, suggesting substantial changes in interfacial organization during adsorption [41]. The randomness at the solid–liquid interface during adsorption may be attributed to the release of hydrated water molecules from both the adsorbent surface and Cr(VI) ions during the adsorption process.

The values of ΔH° are high and positive, implying that the adsorption process was endothermic. The increase in K_d_ with temperature further supports this behavior. The relatively high ΔH° values observed for PUF and mAC/PUF suggest that adsorption may involve interactions between Cr(VI) and CR species and surface functional groups. Positively polarized active sites on the Zr-MOF surface act as active sites capable of coordination with Cr(VI). Furthermore, hydrogen bonding and surface complexation may occur, enhancing the adhesion strength on the material surface. Notable adsorption at high temperature can increase collisions of solution molecules with the adsorbent surface and facilitate faster diffusion into the pores of the adsorbent materials. Thus, increasing the temperature accelerated the movement of adsorption sites and improved accessibility.

The relatively low activation energy indicated that the adsorption process may have been governed primarily by weak surface interactions and diffusion-related phenomena. Incorporation of Zr-MOF and mAC increased the number of active sites and enhanced interactions between the adsorbent surface and pollutants through electrostatic attraction, hydrogen bonding, and surface complexation. Therefore, the adsorption mechanism was likely controlled by a combination of physical adsorption, surface interactions, and mass diffusion rather than just strong chemical bonds [46].

#### 3.3.4. Effect of pH

**Figure 9:**
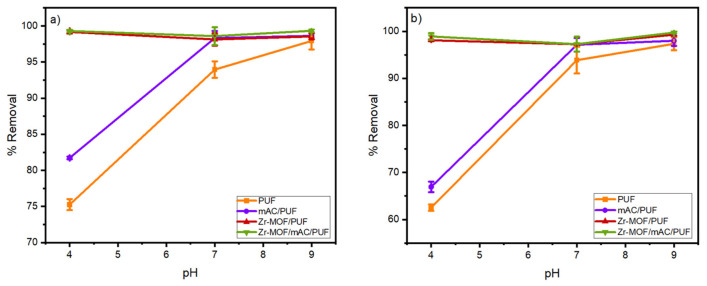
Effect of pH on % removal (**a**) Cr(VI) and (**b**) CR on PUF, mAC/PUF, Zr-MOF/PUF, and Zr-MOF/mAC/PUF adsorbents.

**Figure 10:**
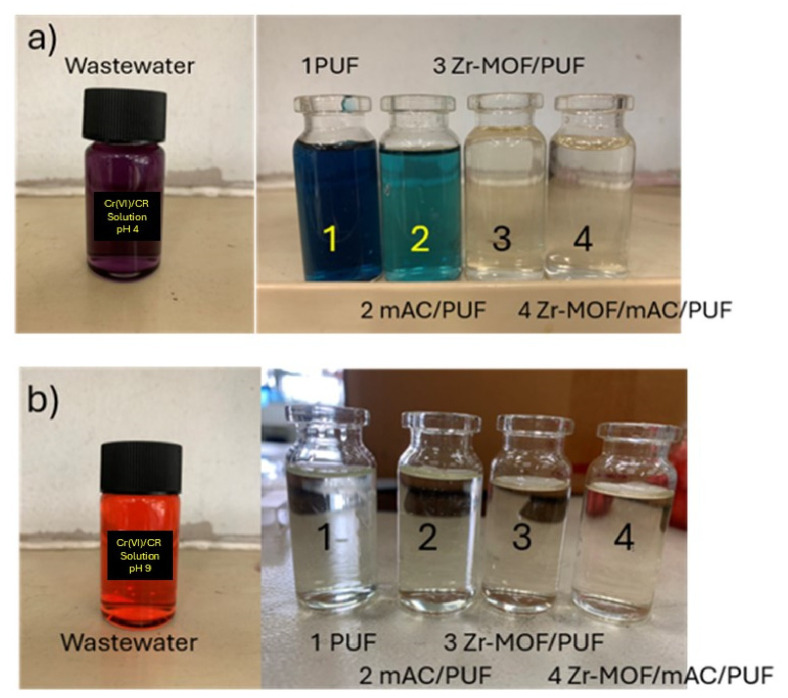
Appearance of wastewater (untreated) and treated with PUF (1), mAC/PUF (2), Zr-MOF/PUF (3), and Zr-MOF/mAC/PUF (4) under 25 °C and 150 rpm for 24 h at different pH: (**a**) pH 4, (**b**) pH 9.

Chromium removal most likely occurs in the acidic pH range of 0.5–4 [41], whereas the typical pH range for dye removal is alkaline, 7–9 [46,47]. This experiment varied the pH from 4 to 9, focusing on the CR range relevant to textile industry applications, from low pH (4, violet) to high pH (above 5.2, red). A synthetic binary Cr(VI)/CR solution was used in this study, with triplicate measurements. The concentration of the Cr(VI)/CR solution (binary solution) was maintained at 50 mg/L, and the experiment was conducted in a shaking incubator at 25 °C and 150 rpm for 24 h. At pH 4, Zr-MOF/PUF and Zr-MOF/mAC/PUF absorbents removed Cr(VI) and CR more effectively than PUF and mAC/PUF, as shown in Figure 9. Figure 10 shows the appearance of wastewater (dark violet at pH 4 and red at pH 9) after treatment at pH 4 and 9 using different absorbents. Zr-MOF/PUF and Zr-MOF/mAC/PUF treated wastewater efficiently at pH 4, and all absorbents absorbed pollutants greatly at pH 9.

In Figure 5, PUF and mAC/PUF are more hydrophobic while Figure 6 exhibits a lower swelling ratio than Zr-MOF/PUF and Zr-MOF/mAC/PUF, which limits mass diffusion of the pollutant to the surfaces. At the same time, Cr(VI) predominantly exists as HCrO_4_^−^, and CR (negatively charged) is adsorbed onto the PUF and mAC/PUF surfaces at pH 4 via electrostatic attraction. In this case, the low removal efficiency of PUF and mAC/PUF at pH 4 might be limited by mass diffusion. A dramatic improvement in removal was observed for PUF and mAC/PUF at pH 9. On the other hand, at pH 4, the Zr-MOF surface carries a positive charge due to free –(COOH) groups, which promotes electrostatic attraction with HCrO_4_^−^ of Cr(VI) and CR [39,48,49,50,51]. Therefore, the removal efficiency of Zr-MOF/PUF and Zr-MOF/mAC/PUF absorbents was high. Please note that both adsorbents also show high hydrophobicity and swelling ratios, allowing water to penetrate the surface and pores. Therefore, they showed a promising Cr(VI) and CR removal efficiency of nearly 100% at pH 9. The investigation of more absorption mechanisms was carried out using kinetic models.

#### 3.3.5. Adsorption Kinetics

The kinetic constant is shown in Table 4. The calculation was performed at pH 4 and 9, referring to the appearance of wastewater in Figure 10. At pH 4, the pseudo-first-order model, PUF, and mAC/PUF had relatively high coefficients of determination for Cr(VI) (R^2^ = 0.9714 and 0.9949) and CR adsorption (R^2^ = 0.9446 and 0.9811), indicating that the adsorption process may involve both physical adsorption and diffusion-controlled mechanisms. In contrast, the Zr-MOF/PUF and Zr-MOF/mAC/PUF composites had lower R^2^ values, suggesting that the pseudo-first-order model could not adequately describe their adsorption behavior. In particular, Cr(VI) adsorption onto Zr-MOF/PUF had poor linearity, implying that the adsorption process was not mainly controlled by simple physisorption.

At pH 9, the pseudo-second-order model provided a much better fit for Cr(VI) and CR adsorption across all absorbents, PUF, mAC/PUF, Zr-MOF/PUF, and Zr-MOF/mAC/PUF, with R^2^ values close to 0.9999. When comparing the q_e_ of PUF and mAC/PUF between pseudo-first order at pH 4 and pseudo-second order at pH 9, the q_e_ increased from 0.8603 (PUF) and 0.8464 mg/g (mAC/PUF) to 1.2354 (PUF) and 1.2373 mg/g (mAC/PUF). The increase in q_e_ might reflect the increased hydrophilicity of the composite materials. At high pH values, a strong negative charge was present on the surface of PUF. At the same time, pollutants also carried a strong negative charge at high pH, creating immediate repulsion. As a result, PUF lost its hydrophobic property, and pollutants diffused into PUF, increasing the removal efficiency. This is an interesting phenomenon in using PUF for binary Cr(VI)/CR wastewater treatment, but it needs more investigation.

Zr-MOF/PUF and Zr-MOF/mAC/PUF performed better than PUF and mAC/PUF absorbents at pH 4 and 9. At pH 4, the functional groups of Zr-MOF, such as -OH, -COOH, and -NH, generated a positive charge at active sites on the surface of the adsorbents, while the pollutants carried a negative charge. The higher adsorption of Zr-MOF/PUF and Zr-MOF/mAC/PUF indicated strong surface interactions between the active sites of the adsorbent and the pollutants. At a high pH of 9, the lower concentration of H^+^ minimized competition with the target ions, thereby enhancing electrostatic attraction and complexation between the adsorbate and the surface sites, which resulted in improved adsorption efficiency [40,52]. Some reports noted a shift of the pKa of UiO-66-(COOH)_2_ to lower values at alkaline pH due to an increase in the number of carboxylate groups in the linker molecule. This occurred because of the negative inductive effect (-I effect) of the -COOH groups and a strong electron-withdrawing group [50,53]. As a result, the partial charges of the Zr^4+^ ions decreased slightly, which led to a slight decrease in the acidity of the coordinating -OH and H_2_O groups. Consequently, the removal efficiency of Zr-MOF remained high.

### 3.4. Regeneration

Regeneration tests were conducted to assess the potential reuse of the adsorbent material. The composite material, Zr-MOF/mAC/PUF, was washed with 0.1 mol/L NaOH for 15 min and then dried at 60 °C for 12 h before reusing. NaOH is an effective regenerant for desorbing Cr(VI) and CR from the used adsorbents. The hydroxyl ion of NaOH creates a negative charge on the adsorbent, while Cr(VI) and CR are anionic pollutants. The strong electrostatic repulsion pushes the pollutants and adsorbent apart. The objective of the present work was to evaluate preliminary reusability rather than to optimize regeneration conditions. Reuse and regeneration were performed for 6 cycles to evaluate sample stability (SEM image in Figure 2 and FTIR profiles in Figure 3). The adsorption efficiency of each cycle was used to analyze the stability and durability of the material [54].

**Figure 11:**
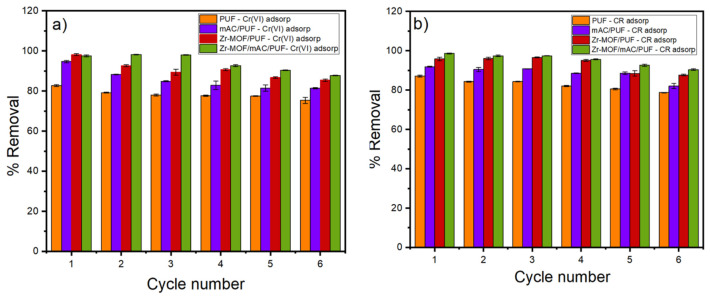
Reusability of composite materials: (**a**) Cr(VI) removal; (**b**) CR removal.

Based on the results, all the materials maintained high efficiency over several cycles, with slight decreases due to surface changes and residual adsorbates. For Cr(VI) removal, the adsorption efficiency of PUF decreased from 81.55% in the first cycle to 73.23% after the sixth cycle (Figure 11a), reflecting the limited number of active sites on the PUF structure. The mAC/PUF materials had higher stability, with efficiency decreasing from 94.26% to 82.33%, as mAC provided additional functional groups for metal binding. Zr-MOF coated onto PUF and mAC/PUF had similar adsorption efficiencies, with initial values of 98.59% and 98.72%, respectively. Zr-MOF/mAC/PUF remained at 85.94% and 88.33% after six cycles for Cr(VI) adsorption, indicating strong structural stability and firm attachment of Zr-MOF to the foam matrix, preserving porosity and active binding sites. A similar trend was observed for CR adsorption, as shown in Figure 11b. After six regenerations and reusability, Zr-MOF/mAc/PUF had the best stability, with a reduction from 98.14% to 90.59%. The order of stability from the highest to lowest was Zr-MOF/mAC/PUF, Zr-MOF/PUF, mAC/PUF, and PUF. Adding mAC and Zr-MOF not only improved the adsorption performance but also improved the stability of the materials.

## 4. Conclusions

This study focused on synthesizing and developing a PUF matrix with mAC added and coated with Zr-MOF via a hydrothermal process to enhance adsorption performance for a binary solution. Other research [53] focused on adsorption in single-material systems, which failed to accurately reflect the actual behavior of industrial wastewater. However, the current research studied binary Cr(VI) and CR systems, which were more complex and commonly found in the textile and tanning industries. Our research achieved adsorption efficiencies exceeding 90% across multiple pH ranges. Based on the SEM results, the incorporation of mAC and Zr-MOF increased surface roughness. At the same time, FTIR confirmed chemical bonding between PUF and the additives and provided evidence that Zr-MOF remained on the surface of the PUF and mAC/PUF samples after adsorption under alkaline conditions. TGA characterization indicated that Zr-MOF/mAC/PUF had the highest thermal stability compared to other absorbent materials. Zr-MOF/mAC/PUF exhibited hydrophilic properties and a high swelling capacity. For the kinetic model investigation, it was found that PUF increased hydrophilicity at higher pH, thereby increasing removal efficiency. At higher pH, all adsorbents performed with high removal efficiency. Zr-MOF/mAC/PUF was the best due to surface complexation with zirconium sites, hydrogen bonding, and π–π interactions. The isotherm showed that the Zr-MOF/mAC/PUF adsorption behavior was a good fit to the Freundlich isotherm and pseudo-second-order kinetic models, but with low activation energy. This indicated that the adsorption was endothermic and driven by electrostatic interactions and mass diffusion control. Furthermore, Zr-MOF/mAC/PUF was reusable at least 6 times, with a 7.55% reduction in removal efficiency from 98.14 to 90.59%. However, further investigation suggested the use of a non-linear isotherm to find a better model for heterogeneous surface adsorption and further investigation of increasing the hydrophilicity of PUF under highly alkaline conditions.

## Figures and Tables

**Table 1:** Thermal degradation factor for TGA of PUF, mAC/PUF, Zr-MOF/PUF, and Zr-MOF/mAC/PUF composites.

Sample	Temperature (°C)					
	T_d5_ (°C)	T_d10_ (°C)	T_d50_ (°C)	Weight Loss 1st Step (300–350 °C) (wt%)	Weight Loss 2nd Step (351–400 °C) (wt%)	Weight Loss 3rd Step (450–600 °C) (%)
PUF	271.00	315.00	400.00	21.64	61.97	-
mAC/PUF	255.00	308.00	402.00	18.39	62.44	-
Zr-MOF/PUF	201.00	321.00	414.00	15.68	51.55	11.59
Zr-MOF/mAC/PUF	116.00	308.00	409.00	14.30	45.74	14.43
Zr-MOF	112.29	144.33	614.00	20.98	10.65	25.18

**Table 2:** Calculated parameters for the adsorption isotherm of Cr(VI) and CR onto PUF, mAC/PUF, Zr-MOF/PUF, and Zr-MOF/mAC/PUF composites.

Solution /Isotherm	Cr(VI)					CR			
	Constant	PUF	mAC/PUF	Zr-MOF/PUF	Zr-MOF/mAC/PUF	PUF	mAC/PUF	Zr-MOF/PUF	Zr-MOF/mAC/PUF
Langmuir	q_max_ (mg/g)	0.4749	0.4879	0.6729	1.7864	0.6695	1.7631	0.7870	10.0684
	K_L_ (L/mg)	2.2636	2.5381	−1.0127	−2.4761	1.2394	0.5015	−1.6398	0.1367
	R_L_	114.1831	127.9094	−49.6355	−122.8067	62.9709	26.0762	−80.9911	7.8389
	R^2^	0.9883	0.9472	0.1613	−0.2545	0.9994	0.9928	0.0321	0.9115
Freundlich	K_f_ (L/mg)	0.3421	0.3612	2.4817	2.6642	0.3510	0.5323	1.6853	1.3186
	n	0.4163	0.4673	1.7731	1.1896	0.3150	0.2373	1.4620	0.3743
	R^2^	0.9881	0.9703	0.5677	0.7664	0.8866	0.2218	0.7607	0.9784

**Table 3:** Thermodynamic parameters of Cr(VI)/CR adsorption onto PUF, mAC/PUF, Zr-MOF/PUF, and Zr-MOF/mAC/PUF composites.

Solutions			Cr(VI)					CR			
Adsorbents	Temperature (°C)	Kd	Gibbs Free Energy ∆G° (kJ/mol)	Enthalpy ∆H° (kJ/mol)	Entropy ∆S° (J/K/mol)	Activation Energy E_a_ (kJ/mol)	Kd	Gibbs Free Energy ∆G° (kJ/mol)	Enthalpy ∆H° (kJ/mol)	Entropy ∆S° (J/K/mol)	Activation Energy E_a_ (kJ/mol)
PUF	298	2.8857	−2.6256			2.4859	1.6526	−1.2447			2.5212
	308	7.5179	−5.1657			2.5690	4.8140	−4.0242			2.6043
	318	11.1065	−6.3652			2.6522	4.9524	−4.2298			2.6875
				53.3160	188.4284				43.6626	152.0381	
mAC/PUF	298	4.4026	−3.6722			2.4850	2.1723	−1.9220			2.4464
	308	13.3678	−6.6395			2.5681	8.8814	−1.9865			2.5296
	318	25.8421	−8.5978			2.6512	8.6899	−5.7164			2.6127
				69.8867	172.5487				54.0036	185.75	
Zr-MOF/PUF	298	103.0268	−11.4835			2.4776	56.4713	−9.9938			2.4780
	308	66.5676	−10.7504			2.5608	160.2903	−13.0007			2.5612
	318	118.0476	−12.6140			2.6439	171.4138	−13.6002			2.6443
				49.2920	153.7176				44.1407	182.92	
Zr-MOF/mAC/PUF	298	130.5789	−12.0706			2.4772	74.7576	−10.6889			2.5234
	308	85.2069	−11.3825			2.5604	207.3333	−13.6597			2.6065
	318	144.8824	−13.1556			2.6436	237.0952	−14.4578			2.6896
				3.6851	51.5834				45.8301	190.7980	

**Table 4:** Kinetic constants of Cr(VI) and CR adsorption on composites at pH 4 and pH 9, according to pseudo-first-order and pseudo-second-order reactions.

Solutions /Isotherm		Cr(VI) pH 4				CR pH 4			
	Constant	PUF	mAC/PUF	Zr-MOF/PUF	Zr-MOF/mAC/PUF	PUF	mAC/PUF	Zr-MOF/PUF	Zr-MOF/mAC/PUF
Pseudo-first order	K_1_ (min^−1^)	0.0016	0.0015	0.2004	0.0047	0.0024	0.0014	0.0050	0.0061
	q_e_ (g/mg)	0.8603	0.8464	1.2282	0.0634	1.0196	0.9671	0.1193	0.1277
	R^2^	0.9714	0.9949	0.5556	0.1819	0.9446	0.9811	0.4815	0.6150
Pseudo-second order	K_2_ (min^−1^)	0.0037	0.0038	0.5700	1.0100	0.0017	0.0027	1.1800	2.0800
	q_e_ (mg/g)	1.2787	1.1401	1.2390	1.2412	1.2084	1.3529	1.2289	1.2325
	R^2^	0.7330	0.8059	0.9999	0.9999	0.0391	0.4127	0.9999	0.9999
Solutions /Isotherm		Cr(VI) pH 9				CR pH 9			
	Constant	PUF	mAC/PUF	Zr-MOF/PUF	Zr-MOF/mAC/PUF	PUF	mAC/PUF	Zr-MOF/PUF	Zr-MOF/mAC/PUF
Pseudo-first order	K_1_ (min^−1^)	0.0197	0.0254	0.0060	0.014	0.0340	0.2210	0.0106	0.0069
	q_e_ (g/mg)	1.1259	1.5932	0.1587	0.2572	1.1959	1.7363	1.8942	3.7009
	R^2^	0.9836	0.9595	0.5903	0.7778	0.9529	0.9388	0.6101	0.6969
Pseudo-second order	K_2_ (min^−1^)	1.53 × 10^13^	1.53 × 10^13^	1.50 × 10^13^	1.52 × 10^13^	1.54 × 10^13^	1.54 × 10^13^	1.55 × 10^13^	1.56 × 10^13^
	q_e_ (mg/g)	1.2354	1.2373	1.2235	1.5226	1.2390	1.5412	1.2445	1.2479
	R^2^	0.9999	0.9999	0.9999	0.9999	0.9999	0.9999	0.9999	0.9999

## Data Availability

The original contributions presented in this study are included in the article. Further inquiries can be directed to the corresponding authors.
